# Social isolation in rats: Effects on animal welfare and molecular markers for neuroplasticity

**DOI:** 10.1371/journal.pone.0240439

**Published:** 2020-10-27

**Authors:** Veronica Begni, Alice Sanson, Natascha Pfeiffer, Christiane Brandwein, Dragos Inta, Steven R. Talbot, Marco Andrea Riva, Peter Gass, Anne Stephanie Mallien

**Affiliations:** 1 Department of Pharmacological and Biomolecular Sciences, University of Milan, Milan, Italy; 2 Department of Psychiatry and Psychotherapy, Central Institute of Mental Health, Medical Faculty Mannheim of Heidelberg University, Mannheim, Germany; 3 Department of Psychiatry (UPK), University of Basel, Basel, Switzerland; 4 Institute for Laboratory Animal Science, Hannover Medical School, Hannover, Germany; Radboud University Medical Centre, NETHERLANDS

## Abstract

Early life stress compromises brain development and can contribute to the development of mental illnesses. A common animal model used to study different facets of psychiatric disorders is social isolation from early life on. In rats, this isolation can induce long-lasting alterations in molecular expression and in behavior. Since social isolation models severe psychiatric symptoms, it is to be expected that it affects the overall wellbeing of the animals. As also promoted by the 3Rs principle, though, it is pivotal to decrease the burden of laboratory animals by limiting the number of subjects (reduce, replace) and by improving the animals’ wellbeing (refine). The aim of this study was therefore to test possible refinement strategies such as resocialization and mere adult social isolation. We examined whether the alternatives still triggered the necessary phenotype while minimizing the stress load on the animals. Interestingly, we did not find reduced wellbeing-associated burrowing performance in isolated rats. The hyperactive phenotype seen in socially isolated animals was observed for rats undergoing the adult-only isolation, but resocializing ameliorated the locomotor abnormality. Isolation strongly affected markers of neuroplasticity in the prefrontal cortex independent of timing: mRNA levels of *Arc*, *Bdnf* and the pool of *Bdnf* transcripts with the 3’ long UTR were reduced in all groups. *Bdnf* splice variant IV expression was reduced in lifelong-isolated animals. Some of these deficits normalized after resocialization; likewise, exon VI *Bdnf* mRNA levels were reduced only in animals persistently isolated. Conversely, social deprivation did not affect the expression of *Gad67* and *Pvb*, two GABAergic markers, whereas changes occurred in the expression of dopamine *d1* and *d2* receptors. As adult isolation was sufficient to trigger the hyperactive phenotype and impaired neuroplasticity in the prefrontal cortex, it could be a candidate for a refinement strategy for certain research questions. To fully grade the severity of post-weaning social isolation and the alternatives, adult isolation and resocialization, a more profound and multimodal assessment approach is necessary.

## Introduction

Chronic stress is a major environmental risk factor for the occurrence of psychiatric disorders. Especially early life stress, such as maternal separation or social isolation, compromises brain development, and can contribute to mental illness [1, 2]. Deficits in the social environment during neurodevelopment are relevant e.g. in autism spectrum disorder, attention-deficit/hyperactivity disorder, and schizophrenia [3, 4].

It has been proposed that social experiences and relationships may influence dopaminergic functionality [5, 6]. For instance, greater dopaminergic and cortisol responses to a psychological stressor were observed in young adults that reported low parental care when compared to controls [7], suggesting that early-life experiences may have a deep impact on systems implied in stress response. Corroborating this notion, exposure of children to abuse or unstable family situations is linked with elevated dopaminergic functionality in the striatum at adulthood [8], as well as with imbalance in excitatory/inhibitory neurotransmitters, which may underlie some cognitive dysfunctions [9]. Moreover, it is thought that childhood trauma might interplay with genetic factors, leading to several alterations–among which decreased levels of neurotrophins, aberrant DNA methylation, hypothalamic-pituitary-adrenal axis dysregulation–potentially culminating in psychotic episodes [10].

Nowadays, social isolation represents a significant health issue. Indeed, since children and adolescents are more sensitive to the detrimental effects of isolation, the COVID-19 containment measures (quarantine and forced isolation) might result in increased anxiety and depression rates among those fragile subjects [11], therefore this historical period demands deeper insights on the tangled consequences of social deprivation.

A common animal model used to study different facets of mental disorders is early-life social isolation in rodents [1]. In rats, this isolation can induce long-lasting alterations in functional connectivity, behavior, and molecular expression [1, 12–14]. The exposure to social deprivation is considered one of the most reliable preclinical models of schizophrenia, as it simulates the core features of mental disorders, e.g. cognitive deficits, alterations in social behavior, hyperactivity, and sensory gating deficits [1]. At the same time, social isolation supposedly impairs the wellbeing of the subjects, as the model aims to evoke psychiatric associated symptoms, which are generally considered disruptive [1]. Yet, it is unknown how much the animals are really burdened by these symptoms.

As in any experimental animal approach, the ethical dilemma between the wellbeing of the individual subject, and the opportunity to gain insight into a scientific question arises. The 3Rs principle states the importance of limiting the number of experiments (reduce, replace) and of improving the wellbeing of subjects (refine) as much as possible while maintaining efficacy. Since rats are social animals, it is advised to keep rats group-housed throughout life, but particularly early in life, when frequency and intensity of social contacts peak during adolescence [15, 16]. During adulthood, social interactions decline without ever diminishing completely [16]. Hence, the consequences of stress exposure are deeply influenced by the period of isolation and the timing thereof. Indeed, a relationship between brain responses to stress and its timing has been proposed. The early life period is very sensitive, characterized by intense brain development, during which external stimuli may influence or interfere with ongoing anatomical and functional changes [17, 18]. Therefore, it is highly likely that challenges that occur during this period may have long-term consequences on brain functions, potentially leading to the outbreak of pathological conditions later in life [19, 20]. During adulthood, by contrast, stress exposure determines more transitory changes in brain structures and functions. The mature brain possesses a heightened ability to respond to challenges with coping strategies that may ultimately attenuate the consequences of such insults, even though chronic exposure may still be harmful to the overall health [17, 21, 22].

Therefore, one way of refining social isolation experiments in terms of animal welfare could be the isolation of subjects in the less sensitive adulthood phase.

Another idea is to resocialize the subjects during adulthood. This could also result in alleviation of some symptoms, while others may remain stable. Evidence from former resocialization studies is inconsistent in this respect [23]. Moreover, housing the rats in groups again after former isolation is often accompanied by aggressive behavior, which could inflict even more welfare concerns [24, 25]. In severe cases, it might become necessary to separate subjects from the group permanently. This, in turn, leads to the exclusion of the animal from the experiment as this repeated social isolation may confound the previous effects and abolishes the comparability of subjects and simultaneously acts against the idea of reducing animal numbers (3Rs).

The aim of this study was to assess the effect of resocialization and adult social isolation as possible refinement strategies for the social deprivation model. We intended to evaluate whether these strategies would still elicit isolation-associated hyperlocomotion and influence molecular parameters, while alleviating the burden on the rats.

The hyperlocomotion is an early onset and robust observation in isolation reared rats and is suggested as a criterion for the so-called isolation syndrome [1, 26]. It is detectable with simple means and is therefore frequently published and shows translational relevance since early-life deprived children have been associated with hyperlocomotion as well [27, 28]. To evaluate a wellbeing-associated parameter, we investigated the burrowing behavior. Burrowing is part of the natural behavioral repertoire of small rodents and supposedly has a self-rewarding component [29–31]. Reduction of this innate and spontaneous behavior can indicate welfare impairments in rodents [30, 32, 33]. It has been associated with many different aversive conditions including with hippocampal damage, epilepsy, stress-induced anhedonia, and/or lack of motivation [34–38].

At the molecular level, the exact mechanisms associated with the isolation-induced behavioral phenotypes are still unclear, although synaptic functions appear to be affected by social deprivation, coinciding with impairments of several neurotransmitters [1]. Impaired neurogenesis, long-term potentiation, and neuroplasticity were observed following isolation rearing conditions [1, 39–41]. Moreover, isolated animals showed hyperactivity of the dopamine system paralleled by reductions of serotonin functioning and reduction of parvalbumin positive gamma-amino-butyric acid (GABA) interneurons [1, 37, 38, 42, 43]. GABAergic transmission is thought to be implicated in cognitive activity and plays an important role in the brain response to stressors [44]. GABAergic dysfunctions are thought to be correlated with cognitive impairment, which is a core symptom of schizophrenia [45]. Similarly, dopamine is relevant for cognitive and emotional functions [46].

Therefore, we assessed the modulation of the expression of brain-derived neurotrophic factor (*Bdnf*), the immediate early gene (IEG) activity-regulated cytoskeleton-associated protein (*Arc*) and the postsynaptic density protein 95 (*Psd95*) involved in learning and memory processes, synaptic plasticity and brain connectivity. Additionally, two GABAergic markers, the glutamic acid decarboxylase 67 (*Gad67*) and parvalbumin (*Pvb*) were analyzed. Lastly, we measured the mRNA levels of the dopamine receptors D1 and D2. The analyses were conducted in the prefrontal cortex (PFC), a brain region highly relevant for the development of psychiatric disorders. Although the role of social behaviors for the development of PFC has been demonstrated [16], there are only a few data on the modulation in this area after a social isolation paradigm [47, 48].

## Materials and methods

### Animals

The subjects of the study were wildtype male Lister Hooded rats, bred and maintained in the animal facility of the Central Institute of Mental Health, Mannheim, Germany. The dark/light cycle of the husbandry was 12h dark/light with lights on at 6 am. Room temperature and humidity were set to 22±1°C and 45±5%. Olfactory, visual, and auditory contact between the cages was not limited, while social interaction with the experimenter was limited to the handling during the weekly cage change. Water and food pellets (LasQCdiet Rod16-H, LasVendi GmbH, Soest, Germany) were available *ad libitum*. The type III (for single-housed rats) and type IV (for group-housed rats) macrolon cages were supplied with Abedd Espen MIDI (ABEDD, Vienna, Austria), but contained no environmental enrichment as suggested in previous publications [1]. All procedures were approved by the German animal welfare authorities (Regierungspräsidium Karlsruhe) and performed strictly according to the regulations of animal experimentation within the European Union (European Communities Council Directive 2010/63/EU).

#### Social isolation paradigm

**Fig 1:**
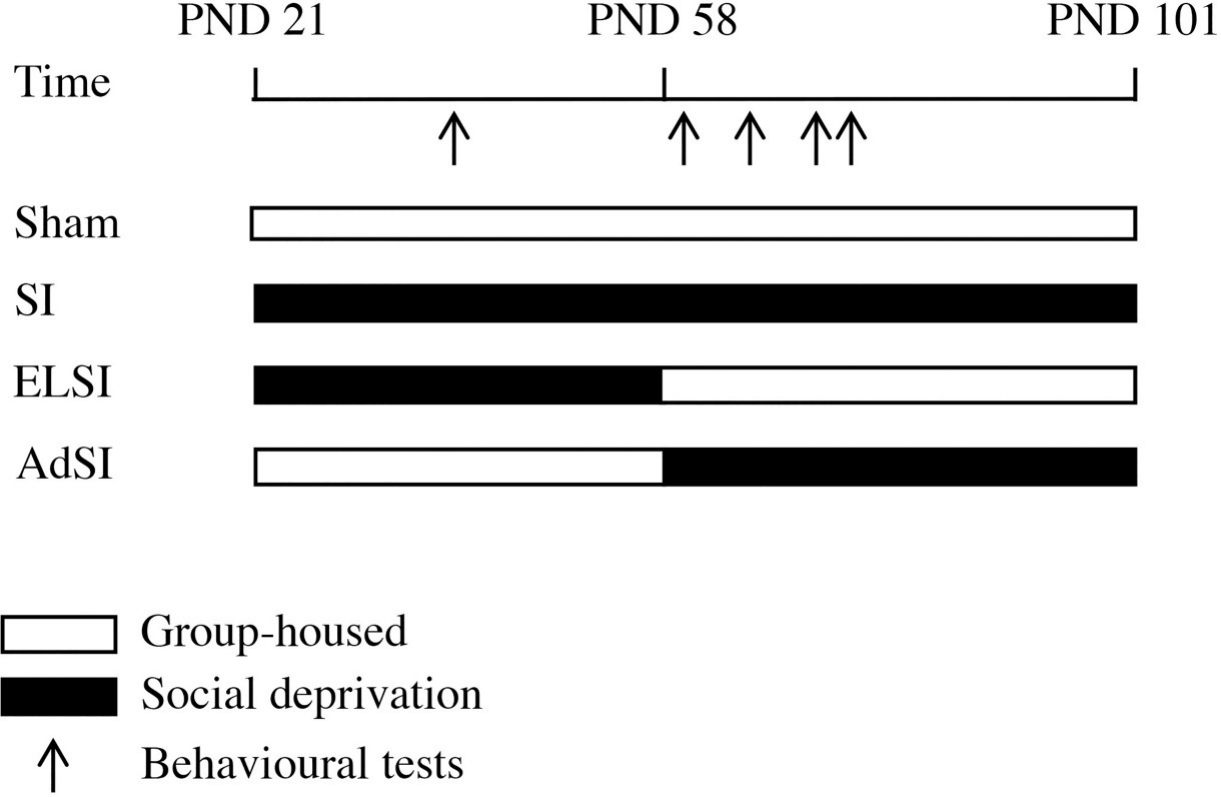
Summary of experimental design. Timeline of stress exposure and behavioral testing in Lister Hooded male rats. Sham = lifelong group-housed animals; SI = lifelong isolated animals; ELSI = animals exposed to social isolation during adolescence; AdSI = animals exposed to social isolation at adulthood.

As shown in Fig 1, the study comprised the analysis of four experimental groups with different exposure to isolation stress: *Social Isolation* (SI) rats were isolated from conspecifics throughout their lifespan (n = 12), *Early Life Social Isolation* (ELSI) rats were only isolated during adolescence (n = 12) and *Adult Social Isolation* (AdSI) rats during adulthood (n = 16). Permanently group-housed rats (Sham) served as controls (n = 16). All animals were weaned at PND 22 ± 1 and pseudo-randomly assigned to group- or solitary housing. The condition change in ELSI and AdSI was performed on PND 58 ± 2. For group housing, four animals were kept per cage. ELSI were resocialized with three littermates. These social partners (n = 36) were not included in the analysis.

### Behavioral analysis

**Fig 2:**
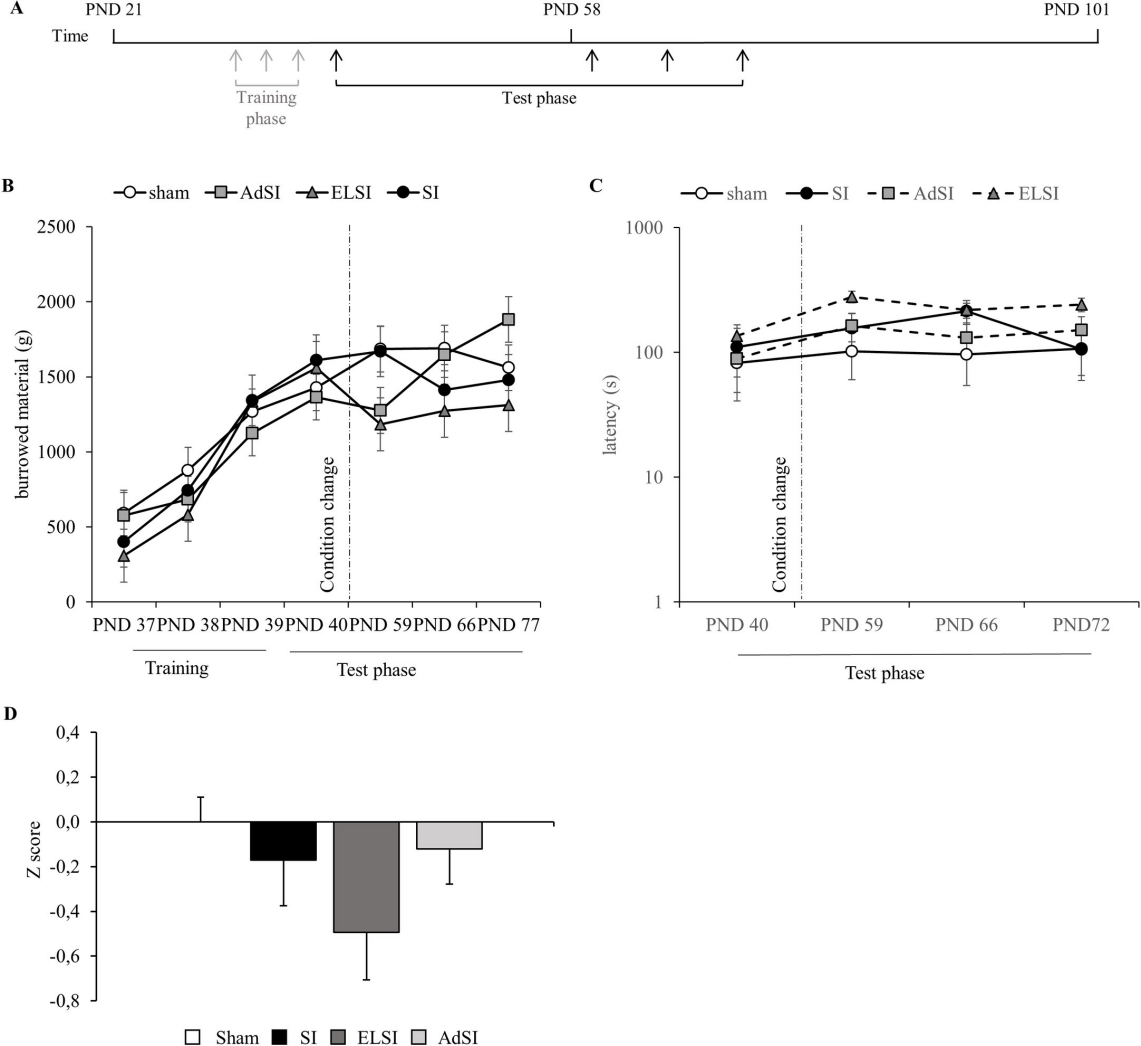


All experiments were conducted approximately one hour after the beginning of the light phase and in separate experimental rooms. Acclimatization to the experimental rooms was only necessary for the Open Field (OF) test (15 min). In a training phase during adolescence (PND 37–39) the subjects were familiarized with the burrowing procedure. The burrowing performance was monitored in the course of the project (PND 40, 59, 66, and 72) (Fig 2A) and locomotion was assessed in the OF test on PND 75.

#### Burrowing

Monitoring of burrowing behavior was assessed as previously described in [49]. Briefly, we placed gravel-filled (2.5 kg) plastic burrowing tubes (32 cm long × 10 cm Ø, opening 60 mm above the floor) in Macrolon Type IV cages and determined the latency for the animal to start burrowing and the burrowed material left in the tube after 60 min. Rats were familiarized with the experimental procedure in a preceding training phase. On the first training day, each rat was free to explore an empty cage for 60 min before they had access to an empty tube for an additional 60 min. The procedure on the three subsequent training days already reflects the test condition: after a 60 min habituation to the empty cage, a gravel-filled tube was introduced into each cage. After another 60 mins, the gravel remaining in the tube was weighed and the amount of removed gravel determined. During the training phase, the latency to start burrowing was not recorded.

#### Open Field test

In the OF test the individual rat was placed into an unfamiliar arena (50 x 50 cm) illuminated from above with 15 lux for 60 min and their activity was recorded with a camera suspended above the arena. For analysis, this period was divided into 10 min time bins and the parameters *movement (time spent moving within the maze)*, *total distance moved*, *velocity*, *time spent in the center of the arena (the area more than 10 cm from the walls)* were analyzed using Ethovision XT, Noldus as described before [50, 51]. The tracking software, which calculates the center of the rat. We manually counted the *number of rearings* (vertical movement in which posture was upright and both front paws were lifted from the ground) and voided feces during the test. The test was performed on PND 75.

### Brain tissue collection, RNA isolation, and real-time PCR analyses

On PND 101±2, the animals were sacrificed. The brains were promptly extracted from the skull and the region of interest immediately dissected on ice. Dissected brains were stored at -80°C before RNA extraction. Total RNA was isolated from the prefrontal cortex by single-step guanidinium isothiocyanate/phenol extraction using PureZol RNA isolation reagent (Bio-Rad Laboratories, Italy) following the manufacturer’s instructions, as previously described [22]. Total RNA was analyzed by TaqMan quantitative reverse transcriptase-polymerase chain reaction (qRT-PCR) to determine the levels of mRNA. Samples were run in triplicate as multiplexed reactions using the iScriptTM one-step RT-PCR kit for probes (Bio-Rad Laboratories). Data were analyzed following the ΔΔCt method with a normalizing internal control, namely β-actin. All primers and probes information is listed in S1 Table.

### Statistical analyses

Data were tested for normal distribution using the Shapiro-Wilk test. Burrowing data were not normally distributed. However, while the residuals of the burrowed material were normally distributed after fitting a linear mixed model with the lme4 [52] package in R (burrowing ~ time*condition + (1|ID)), the residuals of the latency data were not normally distributed. Therefore, the latency data were log-transformed and the model was calculated with the nlme package [53], also in R [54] (burrowing ~ time*condition, random = ~1|ID) to obtain suitable residuals. In both calculations ID was considered a random effect. Factors were treated as simple treatment contrasts with the default level in the intercept. Post-hoc analyses were Tukey corrected.

Repeated measures ANOVA was used to analyze the Open Field parameters. Here, post hoc analyses were Bonferroni corrected. We chose single rats as the experimental unit. No data were excluded from the behavioral dataset.

Normalization of data using the z-score method was performed according to [55]. Basically, it indicates how many standard deviations an observation is above or below the mean of a control group. Applying this mathematical tool, we aimed to define an “emotionality” score enhancing the accuracy of the behavioral phenotyping. Accordingly, at a preclinical level, this approach has been documented as supportive in facilitating the behavioral assessment that is based on a set of converging traits rather than by a single one [55]. We applied z-normalization across multiple and complementary behavioral parameters measuring wellbeing, locomotor activity, and anxiety-like traits. Z-scores in the burrowing task were calculated for each animal using the normalization of *burrowed material* values of each training and testing day. In the Open Field, for each animal the z-score of locomotion activity was calculated using the normalization of *velocity*, *total distance moved*, and *total movement* values. Similarly, z-scores of anxious behavior in the OF were calculated for each animal using the normalized *number of feces*, *number of rearings*, *time spent in the center*, and “distance to walls” values. As an example, z-scores assessing locomotor activity were calculated for each animal using the normalization of velocity, total distance moved, and total movement values.

$$Z\;locomotor\;activity=\frac{(\frac{X-\mu }{\sigma })velocity+(\frac{X-\mu }{\sigma })total\;distance\;moved+(\frac{X-\mu }{\sigma })total\;movement}{number\;of\;parameters}$$

X represents the individual value, whereas μ and σ represent the mean and the standard deviation of sham animals.

Molecular changes produced by isolation were analyzed using univariate ANOVA (one-way ANOVA) followed by Bonferroni post-hoc comparisons, when appropriate. In addition, to evaluate the association between the hyperactive phenotype and the altered gene expression, Pearson product-moment correlation coefficients (*r*) were calculated between the total distance moved (40–60 min) and the mRNA levels of *Bdnf*, *Arc*, *Psd95*, *Gad67*, *Pvb*, *Drd1* and *Drd2*.

A probability level of p<0.05 was considered significant in every test. All analyzes were done with SPSS Statistics software package, Version 25.

## Results

### Social isolation does not induce reduced wellbeing associated burrowing behavior

We used burrowing as a parameter for wellbeing associated behavior. We compared the amount of burrowed material in the preceding training phase and during the tests and found no significant differences between the treatment groups (Fig 2A).

The prominent decrease on PND 59 of the ELSI group was not sufficient to cause statistical differences. An overall time effect (F(6,309) = 9.249, p<0.0001) was detected, but there was no interaction between time and treatment group (Fig 2B). The intercept (F(1,309) = 15.117, p = 0.0001) of the default model was 592.2 g (SE 152.3 g).

The intercept (F(1,157) = 245.787, p<0.001) for the latency to burrow was 53.6 s (SE 1.3 s).We found no differences in the latency to burrow between the conditions, over time or interaction. In comparison of the fixed effects we found that ELSI rats at PND 59 showed a significant increase (p = 0.0304) with an alteration of 2.6 s (SE 1.6 s). No significant differences in z-scores were detected (Fig 2D).

### Isolation-induced hyperactivity is age-independent and can be ameliorated by resocialization

**Fig 3:**
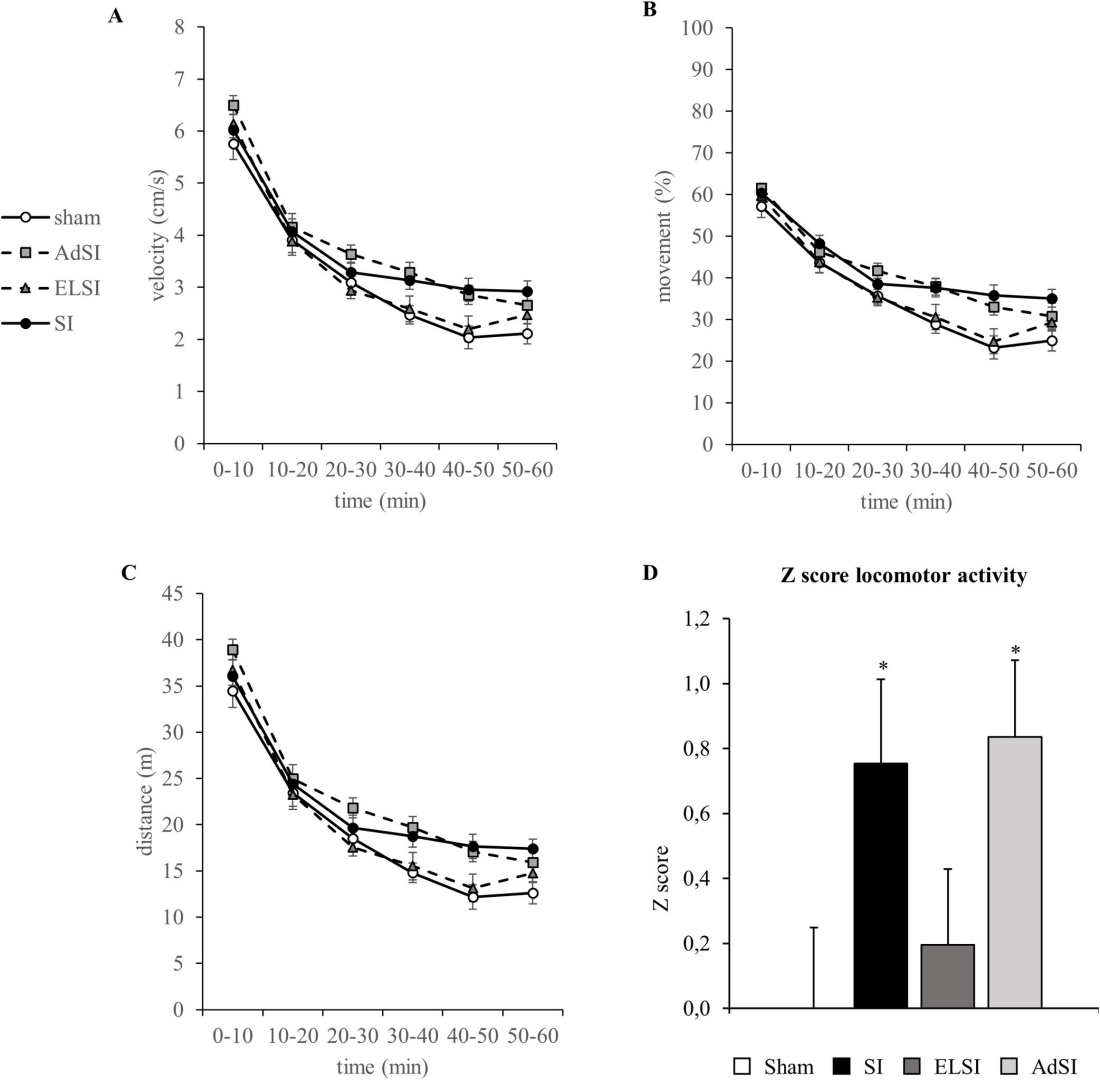
Effects of social isolation on locomotor activity investigated in the Open Field test to assess schizophrenic-like phenotypes. Analyses of velocity in cm/s (A), movement in % (B) and distance moved in m (C) in the Open Field test for all four groups. Normalization of data using the z-score method was performed for *velocity*, *total distance moved*, and *total movement* values using the Sham group as the baseline (D). Each value represents the mean ± SEM. * p<0.05, ** p<0.01 vs Sham animals post-hoc.

Social isolation is known to induce hyperactivity in rats [1]. We detected the locomotor behavior in the Open Field test and observed the progress over time in different parameters (Fig 3A–3C). We found the typical decrease of activity over time for velocity (time F(5,260) = 370.430, p = 5.02E-116), movement (time F(5,260) = 246.846, p = 1.59E-96) and distance moved (F(5,260) = 370.192, p = 5.40E-116). Additionally, we found interactions with the housing condition (distance moved: time*housing F(15,260) = 1.685, p = 0.054; velocity: time*housing F(15,260) = 1.730, p = 0.046; movement: time*housing F(15,260) = 246.846, p = 0.010).

Interestingly, while rats from SI and AdSI showed higher activity, the behavioral outcome from ELSI rats rather resembled the sham controls. We discovered a strong tendency for housing-specific effects (distance moved: housing F(3,52) = 2.716, p = 0.054; velocity: housing F(3,52) = 2.718, p = 0.054), but was only significant for the movement parameter (housing: F(3,52) = 3.372, p = 0.025). Post-hoc analysis revealed increased activity of SI rats, reflected in a statistical trend (p = 0.074, Bonferroni corrected).

The z-score analysis (Fig 3D) further confirmed these results, showing a significant main effect of housing condition (F(3,55) = 2.936, p = 0.042).

**Fig 4:**
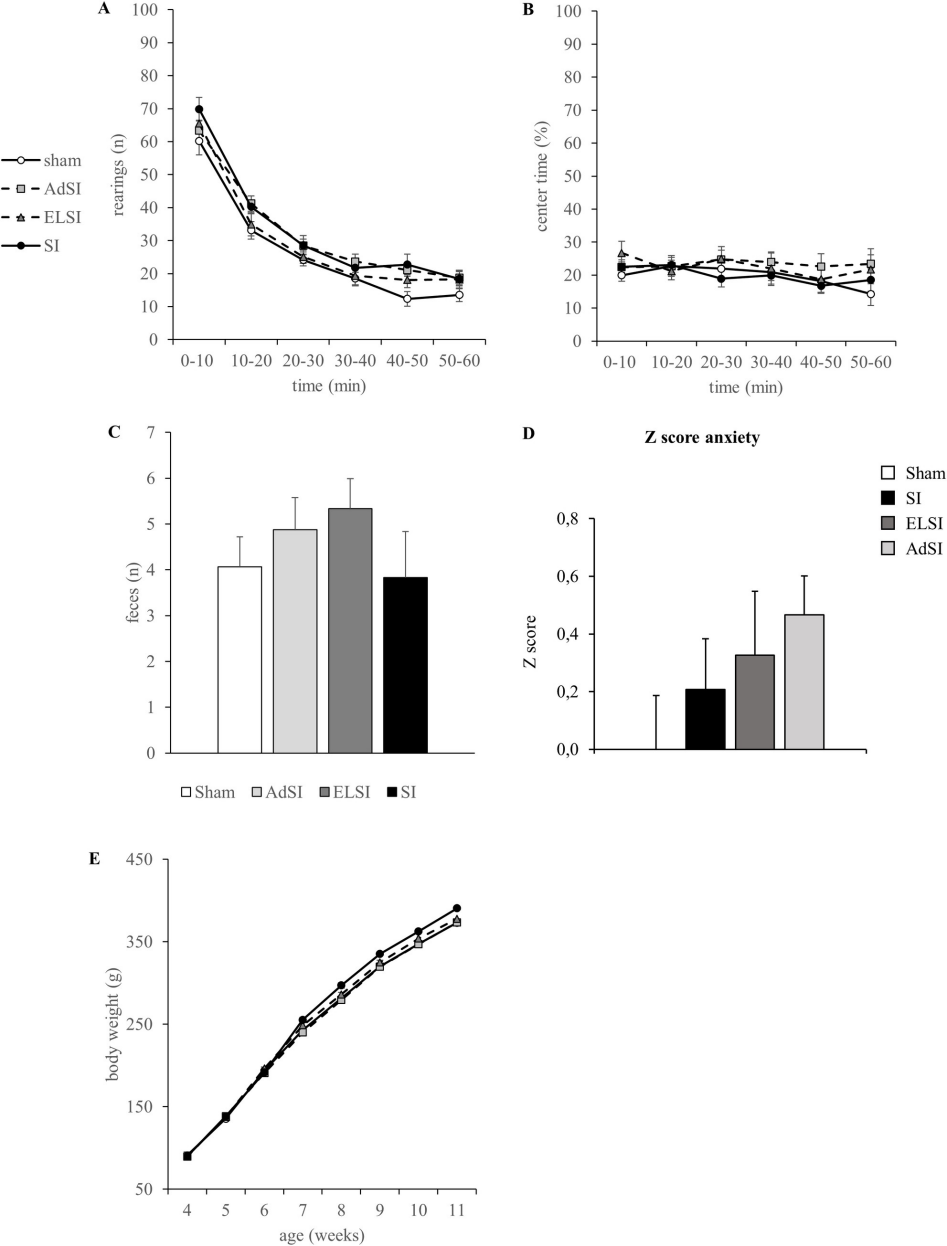
Effects of social isolation on exploration and anxiety in the Open Field. Analysis of the total number of rearings (A), time spent in the center zone (B), and the number of voided feces (C) in the Open Field test. Normalization of data using the z-score method was performed for the *number of feces*, the *number of rearings*, *time spent in the center*, and “distance to walls” values using the Sham group as the baseline (D). Each value represents the mean ± SEM. Body weight development in g over time (E).

The number of rearings in the Open Field test decreased significantly over time (time: F(5,260) = 331.429, p = 5.84E-76), but was not influenced by housing (Fig 4A). The time spent in the center was neither influenced by time nor housing (Fig 4B). All groups voided a comparable number of feces (Fig 4C). There was no significant interaction between time and housing. These data were confirmed by using the z scores approach (Fig 4D), as no effect of housing condition has been observed (F(3,55) = 1.370, p = 0.262).

All rats showed a gain in body weight (time: F(7,371) = 6875.075, p = 2.45E-166), but with different intercepts (time*housing: F(21,371) = 2.568, p = 2.16E-4), which did not result in differences between the housing groups (Fig 4E). While all animals gained weight over time, SI rats showed the highest increase, resulting in a slightly, but insignificantly higher weight.

### Deprivation of social interactions strongly affects neuroplasticity regardless of the timing of stress exposure

#### Analysis of *Bdnf* mRNA expression

To assess the differential impact of social isolation throughout life on neuroplasticity, total *Bdnf* mRNA levels, the pool of *Bdnf* transcripts with the long 3’-UTR that are usually targeted to dendrites, and of two major *Bdnf* isoforms, namely transcript IV and VI, were analyzed in the prefrontal cortex.

**Fig 5:**
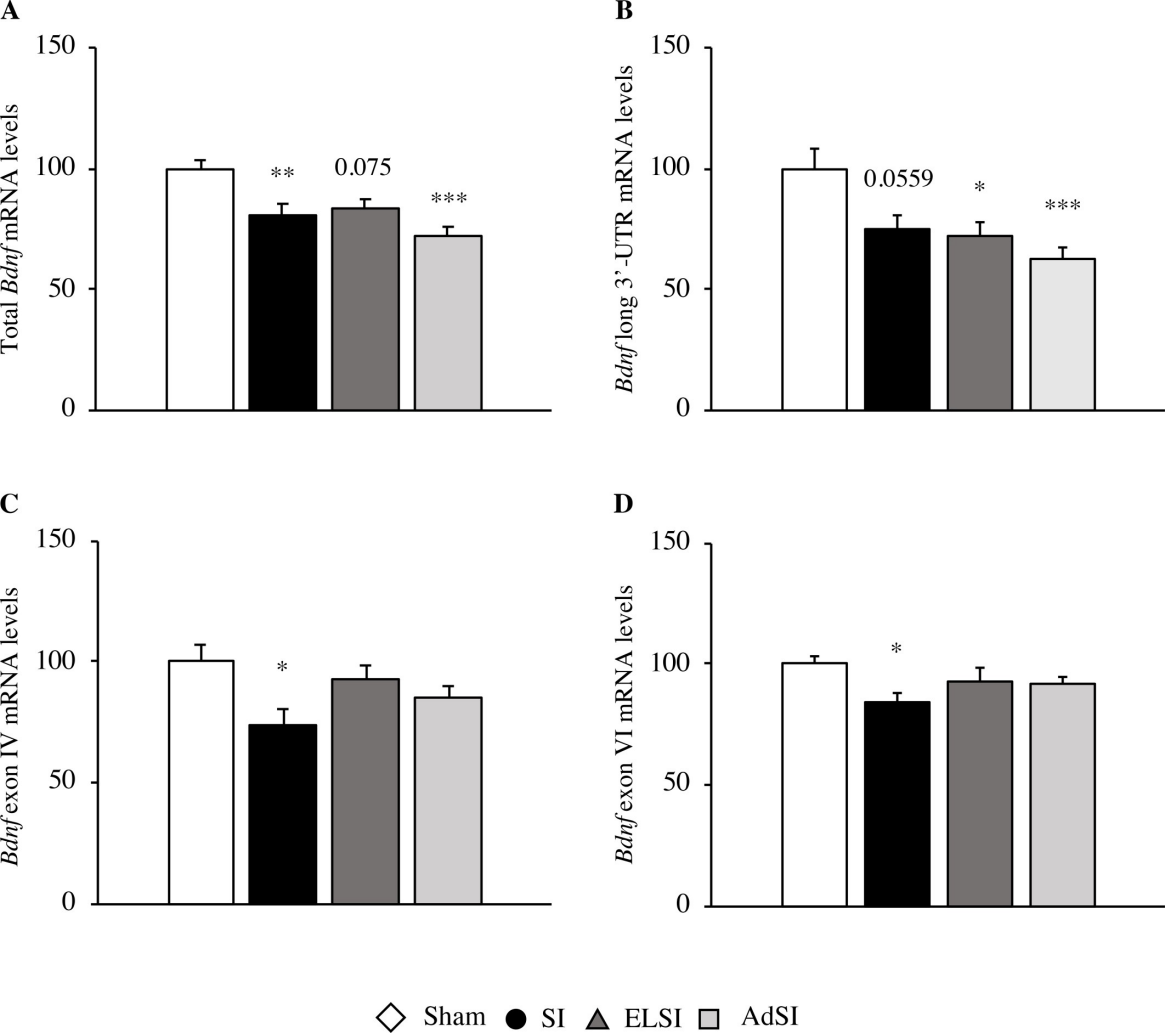
Effects of social isolation on *Bdnf* mRNA expression in the prefrontal cortex of adult Lister Hooded male rats. The mRNA levels of total *Bdnf* (A), *Bdnf* long 3’-UTR (B), *Bdnf* exon IV (C) and *Bdnf* exon VI (D) were analyzed in the prefrontal cortex of male rats that were lifelong group-housed (Sham), lifelong isolated (SI), exposed to social isolation during adolescence (ELSI) or exposed to social isolation during adulthood (AdSI). The data expressed as the % vs. Sham animals set at 100%, are the mean ± SEM. *p<0.05, **p<0.01, ***p<0.001 vs Sham animals post-hoc.

We found that housing conditions determined a significant main effect on the expression of total *Bdnf* mRNA levels within the prefrontal cortex (F(3, 49) = 8.776, p = 0.0001, see Fig 5A). Indeed, animals with lifelong isolation as well as animals which were isolated exclusively during adulthood showed a significant down-regulation of total *Bdnf* mRNA levels, as compared to animals always housed in group (post-hoc: p<0.01 and p<0.001, respectively). Contrastingly, this reduction did not reach statistical significance in animals isolated only during adolescence (p = 0.075).

Similarly, a significant main effect of the rearing condition on the expression of *Bdnf* long 3’-UTR mRNA levels was found (F(3, 52) = 6.750, p = 0.00067, see Fig 5B), with a significant decrease in the expression of *Bdnf* long 3’-UTR within the prefrontal cortex of animals isolated only during adolescence or adulthood (post-hoc: p<0.05 for ELSI and p<0.001 for AdSI), whereas this reduction was barely not statistically significant in lifelong isolated animals (post-hoc: p = 0.0559).

The expression of the two major *Bdnf* isoforms IV and VI were partially divergent from what observed for total *Bdnf* and *Bdnf* long 3’-UTR. As for the expression of *Bdnf* transcripts containing exon IV, we observed a significant main effect of the housing condition (F(3, 54) = 3.302, p = 0.027, see Fig 5C). In detail, animals with a lifelong social deprivation showed decreased exon IV containing-*Bdnf* mRNA levels, as compared to controls (post-hoc: p<0.05), whereas animals isolated exclusively during adolescence or adulthood did not.

In a similar way, data obtained from the analysis of the expression of *Bdnf* transcripts containing exon VI highlighted a significant main effect of housing condition (F(3, 51) = 3.007, p = 0.039, see Fig 5D), with a significant down-regulation of gene expression only in animals lifelong socially deprived as compared to control grouped animals (post-hoc: p<0.05).

**Fig 6:**
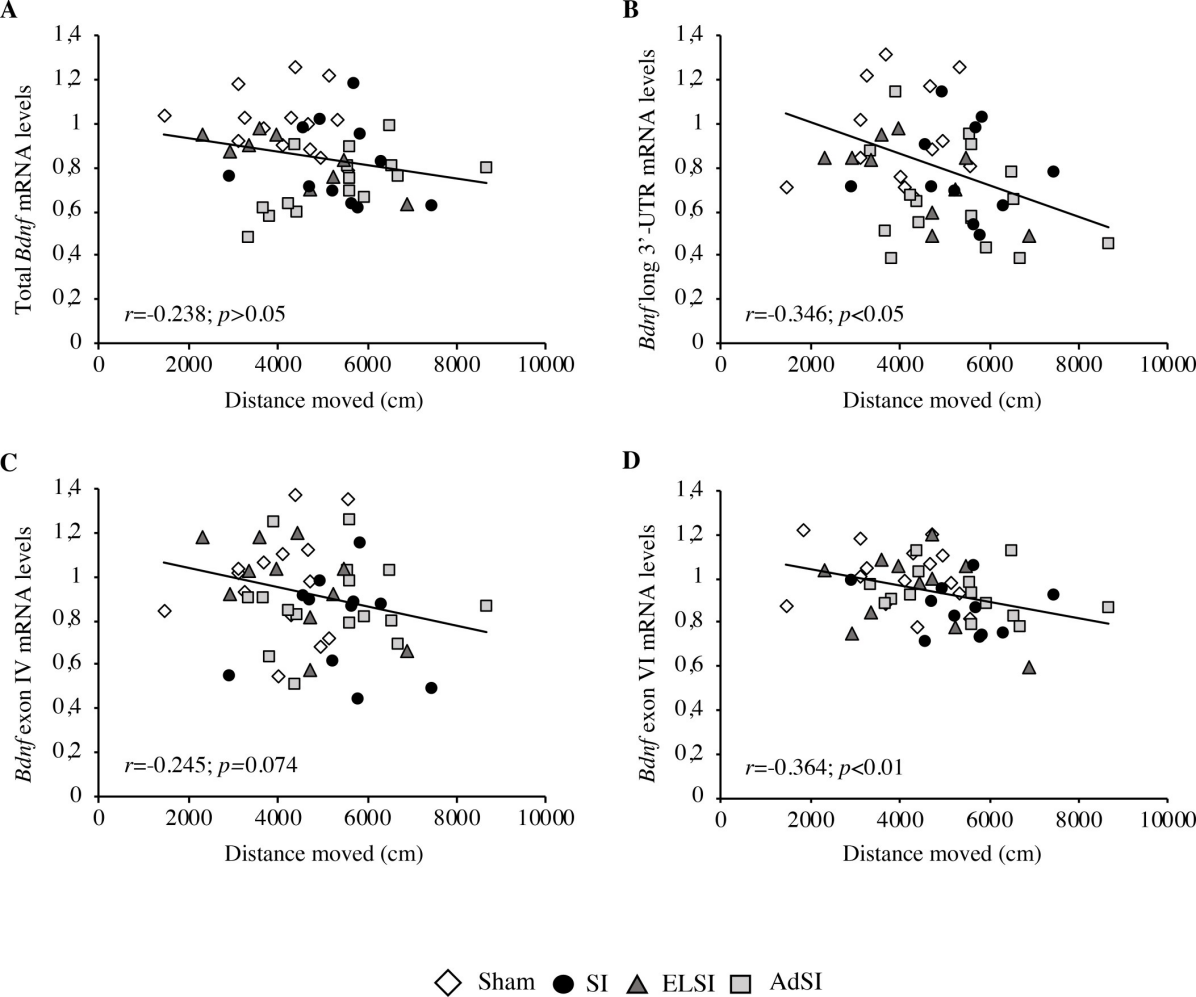
Correlation analyses between the locomotor activity assessed in the OF test and the expression of *Bdnf* within the prefrontal cortex of adult Lister Hooded male rats exposed to social isolation. The correlations between the distance moved in the second part of the OF test (40–60 min) and the mRNA levels of total *Bdnf* (A), *Bdnf* long 3’-UTR (B), *Bdnf* exon IV (C) and *Bdnf* exon VI (D) in the prefrontal cortex of male rats that were lifelong group-housed (Sham), lifelong isolated (SI), exposed to social isolation during adolescence (ELSI) or exposed to social isolation during adulthood (AdSI) were analyzed by Pearson product-moment correlation (r).

In light of these findings, to further investigate if impairments in *Bdnf* expression were associated with the hyperlocomotion observed in the OF test, total *Bdnf*, *Bdnf* long 3’-UTR, *Bdnf* IV transcripts and *Bdnf* VI transcripts mRNA levels were then plotted against the distance moved in the second part of the behavioral test (40–60 min), where the differences appeared most prominent (Fig 6). While no correlation between the distance moved and the expression of total *Bdnf* was found (r = -0.238, p>0.05, Fig 6A), the statistical analysis showed a significant correlation between hyperlocomotion and *Bdnf* long 3’-UTR down-regulation (r = -0.346, p<0.05, Fig 6B) as well as between hyperlocomotion and *Bdnf* VI transcripts down-regulation (r = -0.364, p<0.01, Fig 6D), with a considerable trend toward significance also for the correlation with *Bdnf* IV transcripts expression (r = -0.245, p = 0.074, Fig 6C). Indeed, lower levels of *Bdnf* were associated with higher moved distances in the OF test.

#### Analysis of *Arc* and *Psd95* mRNA expression

Next, we evaluated the levels of the immediate early gene (IEG) Activity Regulated Cytoskeleton Associated Protein (*Arc*) and Post-Synaptic Density Protein 95 (*Psd95*).

**Fig 7:**
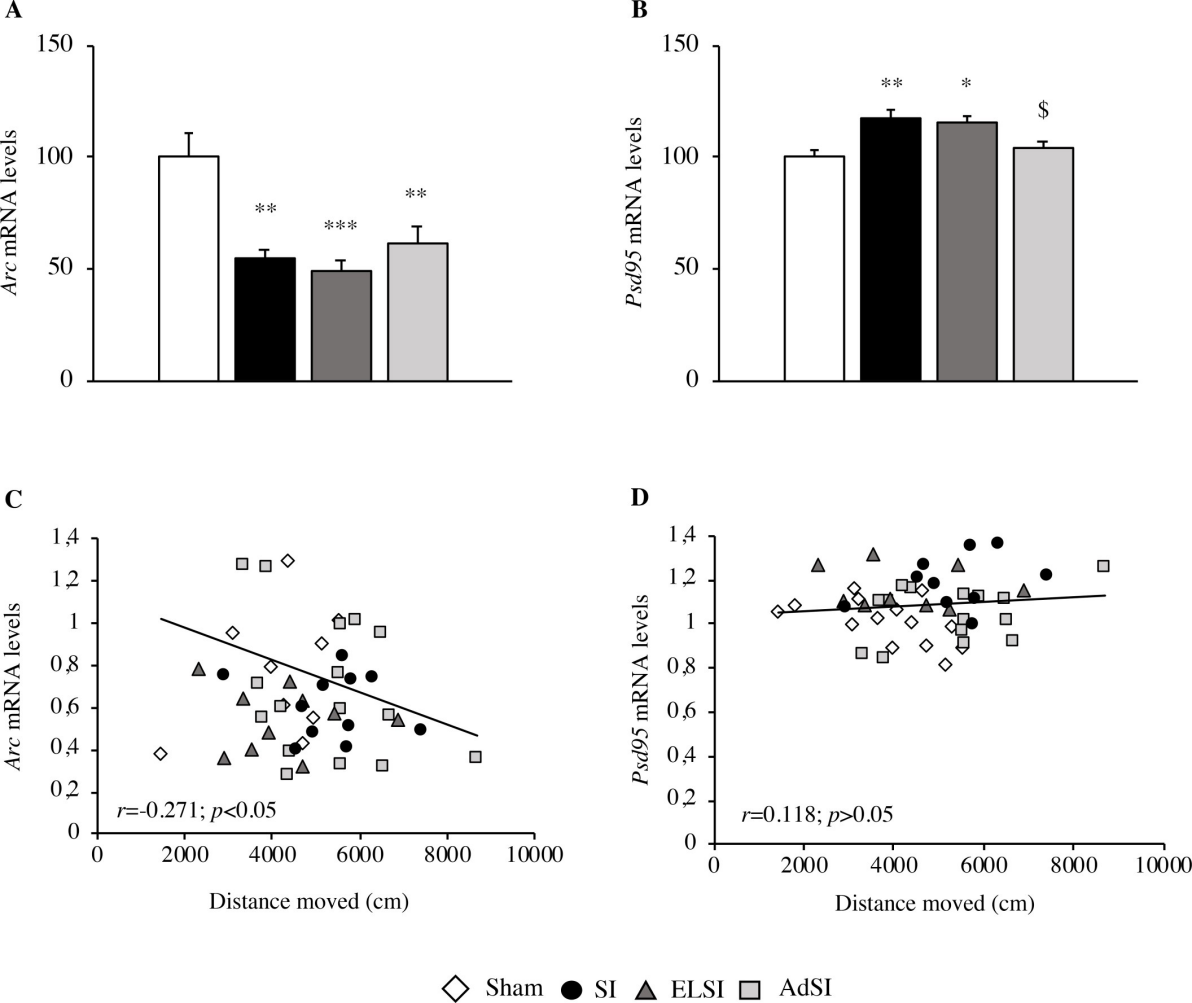
Effects of social isolation on the expression of genes involved in neuroplasticity in the prefrontal cortex of adult Lister Hooded male rats. The mRNA levels of *Arc* (A) and *Psd95* (B) were analyzed in the prefrontal cortex of male rats that were lifelong group-housed (Sham), lifelong isolated (SI), exposed to social isolation during adolescence (ELSI) or exposed to social isolation during adulthood (AdSI). The data expressed as the % vs. Sham animals set at 100%, are the mean ± SEM of at least 9 animals per group. *p<0.05, **p<0.01, ***p<0.001 vs Sham animals; ^$^p<0.05 vs SI animals post-hoc. The correlations between the distance moved in the second part of the OF test (40–60 min) and the mRNA levels of *Arc* (C) and of *Psd95* (D) in the prefrontal cortex of male rats that were lifelong group-housed (Sham), lifelong isolated (SI), exposed to social isolation during adolescence (ELSI) or exposed to social isolation during adulthood (AdSI) were analyzed by Pearson product-moment correlation (r).

We found that housing environment significantly affected *Arc* expression (F(3, 53) = 8.860, p = 0.00008, Fig 7A), resulting in a significant decrease of mRNA levels in the prefrontal cortex of each stressed group, as compared to controls (post-hoc: p<0.01 for SI and AdSI, p<0.001 for ELSI). Furthermore, we observed that the molecular changes in *Arc* expression correlated with the locomotor activity assessed in the OF test. Indeed, there was a negative correlation between the two variables (r = -0.271, p<0.05, Fig 7C), indicating that animals expressing lower *Arc* levels moved more during the second part of the locomotor assessment (40–60 min).

Considering *Psd95* levels, a significant main effect of the housing condition was found (F(3, 48) = 6.889, p = 0.0006, Fig 7B). Conversely to what we observed so far, animals that underwent isolation during adolescence, as well as animals that were always housed in isolation, showed significantly up-regulated mRNA levels in the prefrontal cortex, as compared to controls (p<0.01 for SI and p<0.05 for ELSI). Moreover, animals reared in constant isolation showed a significant increase of *Psd95* also as compared to animals exposed to isolation rearing only during adulthood (p<0.05). A Pearson product-moment correlation coefficient was computed to assess the relationship between *Psd95* levels and distance moved in the second part of the OF test (40–60 min). However, the hyperactivity state did not seem to correlate with the specific modulation of Psd95 mRNA levels (r = 0.118, p>0.05, Fig 7D).

#### Analysis of *Gad67* and *Pvb* mRNA expression

We next evaluated the expression of two γ-aminobutyric acid (GABA) related markers, glutamic acid decarboxylase 67 (*Gad67*), and *Parvalbumin (Pvb)*.

**Fig 8:**
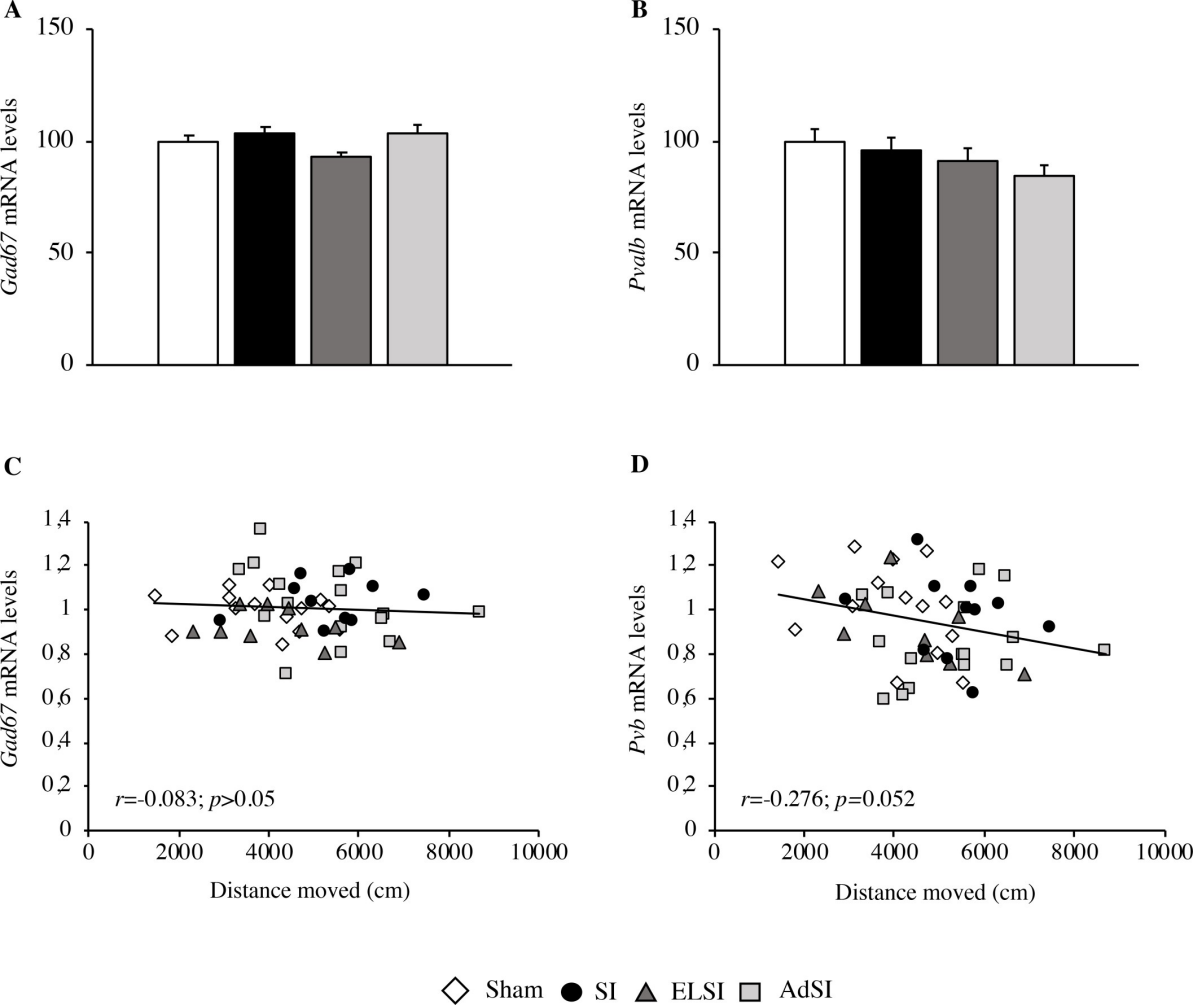
Effects of social isolation on the expression of GABAergic markers in the prefrontal cortex of adult Lister Hooded male rats. The mRNA levels of *Gad67* (A) and *Parvalbumin* (*Pvalb*) (B) were analyzed in the prefrontal cortex of male rats that were lifelong group-housed (Sham), lifelong isolated (SI), exposed to social isolation during adolescence (ELSI) or exposed to social isolation during adulthood (AdSI). The data expressed as the % vs. Sham animals set at 100%, are the mean ± SEM of at least 9 animals per group. The correlations between the distance moved in the second part of the OF test (40–60 min) and the mRNA levels of *Gad67* (C) and *Parvalbumin* (*Pvalb*) (D) in the prefrontal cortex of male rats that were lifelong group-housed (Sham), lifelong isolated (SI), exposed to social isolation during adolescence (ELSI) or exposed to social isolation during adulthood (AdSI) were analyzed by Pearson product-moment correlation (r).

With regard to *Gad67* mRNA expression, we observed no significant main effect of the housing condition (Fig 8A). The lack of a correlation between mRNA levels and locomotor activity assessed during the second part of the OF test (40–60 min) (r = -0.083, p>0.05, Fig 8C) further confirms this.

Although the expression of *Pvb* was not impacted by isolation rearing conditions (Fig 8B), the correlation analysis between the molecular and the behavioral data showed an inverse relationship close to significance (r = -0.276, p = 0.052, Fig 8D). This result indicates that hyperactive animals expressed lower *Pvb* within the prefrontal cortex.

#### Analysis of *Drd1* and *Drd2* mRNA expression

Lastly, we measured the expression of *Drd1* and *Drd2*, genes encoding for dopaminergic receptors subunits D1 and D2, respectively.

**Fig 9:**
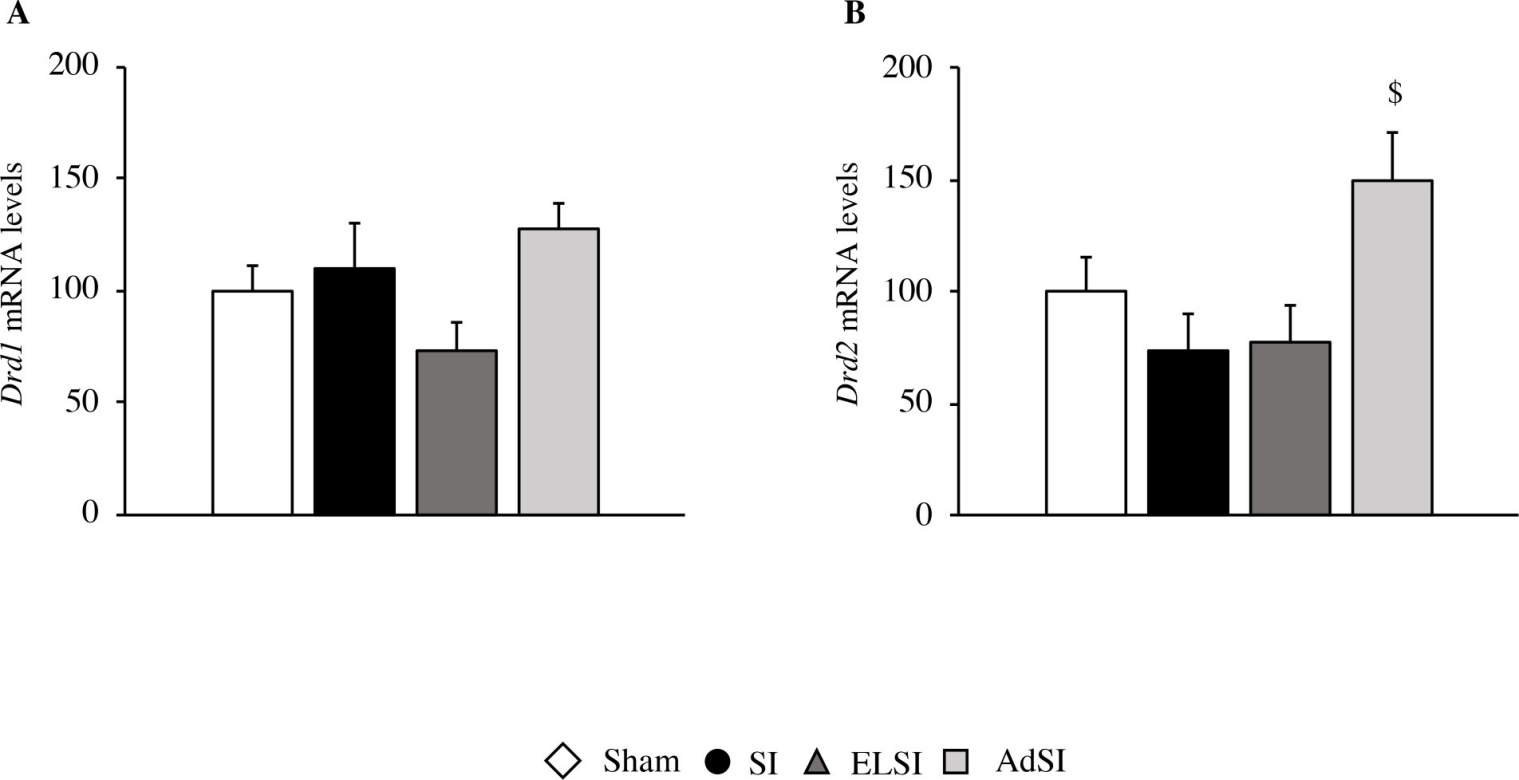
Effects of social isolation on the expression of dopaminergic markers in the prefrontal cortex of adult Lister Hooded male rats. The mRNA levels of *Drd1* (A) and *Drd2* (B) were analyzedanalysed in the prefrontal cortex of male rats that were lifelong group-housed (Sham), lifelong isolated (SI), exposed to social isolation during adolescence (ELSI) or exposed to social isolation during adulthood (AdSI). The data expressed as the % vs. Sham animals set at 100%, are the mean ± SEM of at least 9 animals per group. ^$^p<0.05 vs SI animals.

Considering *Drd1*, we found a substantial trend toward the significance of housing condition (F(3, 49) = 2.653, p = 0.0596, Fig 9A). With respect to *Drd2* mRNA levels, we observed a significant main effect of the housing environment (F(3, 45) = 3.773, p = 0.017, Fig 9B). Indeed, animals isolated only during adulthood show an elevation of *Drd2* expression, which was statistically significant when compared to lifelong isolated animals (post-hoc: *p*<0.05).

Despite these effects, we did not find any correlation between *Drd1* and *Drd2* gene expression and locomotor activity assessed during the second part of the OF test (40–60 min, data not shown).

## Discussion

In this study, we aimed to investigate the effect of social isolation at different developmental stages of male rats, namely during adolescence, young adulthood, or both phases and the effects of resocialization. One reason to investigate this issue was to find potential refinement strategies, which may inflict as little suffering in the subjects as possible, while still being able to investigate the effects produced by social deprivation. To ensure this, we tested the behavioral outcome by assessment of hyperlocomotion, a core behavioral domain for this manipulation. Additionally, we used burrowing behavior as a readout for animal welfare and eventually, we correlated the results with selected molecular changes.

The hyperactivity in a novel environment observed in the Open Field test is typical for the utilized Lister Hooded strain [56–58]. We found that animals, which were socially isolated for their entire life, showed increased locomotor activity, mainly after 40 min within the start of the behavioral task. Similarly, animals isolated only during adulthood displayed a hyperactive phenotype. Contrary, returning to a social context during adolescence normalized the behavioral phenotype. Interestingly, subjects that were isolated at the time of testing showed the typical hyperactivity, independent from the separation status during adolescence. This implies that social isolation acts as an age-independent stressor and that social isolation of rats should be avoided throughout life, whenever possible; not only to minimize confounding factors in scientific readouts but also to warrant animal welfare and to comply with the 3Rs principle of animal research. Similar to previous findings, we found that the resocialized ELSI group did not display hyperactive behavior [56]. However, some behavioral responses cannot be attenuated by social contact after the critical period for social development, e.g. social anxiety, sexual or aggressive interaction [59–62]. Others can be reversed, including approach behavior upon pro-social calls or abnormal habituation to novel environments [63]. The findings on resocialization are inconsistent and appear dependent on different rat strains, sex, or the timing and duration period of social isolation [23]. Typically, rats are isolated after weaning for 2 to 6 weeks and are than resocialized for some time before the experimental onset. This time of resocialization varies highly, from hours to weeks–which of course limits the comparability of the outcomes. However, some studies indicate a critical developmental phase in which for example anxiety, aggression and reduced social interaction and defense behavior remained impaired and could not be recovered by resocialization ([24, 25, 59, 61, 64]; for review read [23]). Other studies show beneficial effects of resocialization on anxiety- and depression-like behavior, as well as aggression and social interaction and response to social ultrasonic calls [63, 65–67]. A direct comparison between adolescent isolation and adult isolation was hardly ever described. Seffer *et al*. Seffer, Rippberger [63] did compare those isolation forms with respect to social approach behavior and found no impairment in isolated adults, but a deficit in resocialized rats [63]. Hence, it is advisable to be aware of whether the isolation-induced effects of interest are distinct after the isolation at different developmental stages.

We also investigated whether the timing of social isolation inflicted a different intensity of severity on the animals’ wellbeing. In principle, social isolation did not derogate the execution of burrowing. Especially during the training and early test phase, no constraints became apparent indicating that the wellbeing of rats was affected by early-onset social isolation.

The AdSI group did not show an increase in burrowing directly after the condition change, but continuously improved afterwards. In contrast, joining a group without previous socialization during adolescence tended to diminish the wellbeing-associated burrowing and increased the latency to start burrowing. The neglect of such "luxury behavior" could be explained by the urge to satisfy more vital needs. The time in the burrowing test may serve as an undisturbed opportunity to sleep, since the rats are alone within the experimental cage. We observed this in several cases. Other than that, we did not detect signs for impaired welfare. Body weight was similar in all treatment groups and we observed no aggressive behavior within the groups, although early life social deprivation has been reported to lead to long-lasting aggressive behaviors [25]. Our data indicate that remaining in social isolation was less stressful than resocialization for adult male rats. Hence, resocialization might not be suitable for refining experiments, although the effects were only tendencies, mild and limited to a short period. It might be advisable to keep this in mind, when performing social alterations in any behavioral experiment.

We looked at *Bdnf*, a marker for psychiatric disorders, which is used as an indicator for wellbeing as well [68]. *Bdnf* is an important player for neuronal development and function, neuroprotection and neuronal plasticity [68, 69]. The latter activity is crucial for adaptation to adverse environmental conditions, which is also associated with the development of neuropsychiatric disorders [70–72]. We found that *Bdnf* expression was markedly reduced in the prefrontal cortex of isolated animals, regardless of the timing of isolation exposure. The *Bdnf* gene is very complex and consists of several splice variants that are localized in different neuronal compartments, mediating synaptic plasticity within each area [73, 74]. Similar to total *Bdnf* expression, we observed that also the levels of the pool of *Bdnf* transcripts with the long 3’-UTR are downregulated within the PFC of isolated animals, without any difference due to the timing of stress exposure. The pool of *Bdnf* transcripts comprising the long 3’-UTR are preferentially targeted to dendrites, contributing to activity-dependent *Bdnf* translation [73]. This may suggest a widespread impairment of the neurotrophic functionality, both in the cell body and in distal dendrites, regardless of the timing of stress exposure. Accordingly, a late deprivation of social contacts, as well as resocialization, seems to be a suitable refinement alternative, leading to a deficiency similar to SI animals. However, it has been suggested that also the specific 5’UTR sequence must be taken into account for dendritic targeting [74]. Accordingly, it has been proposed that *Bdnf* transcripts containing 5’UTR exon I or IV are localized to the cell body and proximal dendrites, while *Bdnf* transcripts containing 5’UTR exon II or VI are found in distal dendrites [74]. Interestingly, we found that exon IV- and exon VI-containing *Bdnf* transcripts were significantly reduced only in lifelong isolated animals. However, *Bdnf* expression patterns differ based on the cell type (neurons or astrocytes) and other minor *Bdnf* transcripts could also play a pivotal role [75, 76]. The adult cortex expresses more exon IV-containing *Bdnf* transcripts than exon VI-transcripts [76]. Stratifying the results at the cellular level, this ratio is confirmed only in neurons, while astrocytes present more exon VI-containing *Bdnf* transcripts in the cortex [75].

A few studies investigating isolation-induced effects on *Bdnf* expression within the PFC found no changes immediately after stress exposure during adolescence [77], while returning to social housing after adolescent social isolation seemed to increase cortical *Bdnf* expression [78]. Similarly, exposure to social deprivation at adulthood induced an up-regulation of *Bdnf* expression in the PFC [79]. Even though our results are in contrast with these findings, they confirm the post-mortem studies in schizophrenia and depressed patients, which showed a consistent decrease of cortical *Bdnf* expression [80]. Furthermore, we found that the isolated animals with reduced *Bdnf* mRNA levels within the PFC were more active in the OF testing. This evidence suggests that the behavioral dysfunction in locomotor activity may be associated with an impairment of neuroplasticity. As both parameters are well-established hallmarks of mental diseases, this correlation strengthens our findings.

The detrimental effects of social deprivation exposure on neuroplasticity were further confirmed by the investigation of *Arc* expression. Similar to BDNF, *Arc* encodes for a multifunctional protein that is fundamental in several processes, comprising synaptic plasticity and structural dendritic spine remodeling [81, 82]. We observed a decrease in *Arc* mRNA expression within the prefrontal cortex of each stressed group, in line with previous studies that found a link between post-weaning isolation and reduced *Arc* levels [83]. A sharp negative correlation between *Arc* levels and locomotor activity further supported that deprivation of social interaction at any time of life can induce a hyperactivity phenotype coupled with impaired neuroplasticity. This finding confirms previously reported data showing how AdSI hyperactive mice also display reduced *Arc* mRNA levels [84]. Nevertheless, further research should investigate isolation at different life-stages to better understand if a link between *Arc* expression and a hyperactivity phenotype is consistent.

Taken together, our data suggest that deprivation of social contacts at any time of life can lead to an overall distress condition of the animals. Hereby, at least as far as *Bdnf* and *Arc* mRNA expression are concerned, we could speculate that the application of social deprivation only during adulthood might be considered as an alternative to long-lasting isolation, inducing the onset of an acute hyperactive behavior as well as a deficit of neuroplasticity-related markers. On the contrary, shifting to a situation of social housing during adulthood normalizes hyperactive phenotypes while still showing molecular impairments, in association with a reduction of wellbeing.

Last, considering the key role of glutamate and GABA in mental illnesses, we investigated two prototypes markers of these systems. We investigated the Postsynaptic Density Protein 95 (PSD95), one of the most abundant proteins of the postsynaptic densities and a key regulator of synaptic transmission and plasticity [85, 86]. Indeed, PSD95 is involved in synaptic maturation by modulating N-methyl-d-aspartic acid receptors (NMDARs) and α-amino-3-hydroxy-5-methyl-4-isox-azoleproprionic acid receptors (AMPARs). Accordingly, a reduction in PSD95 levels has been found in the brain of schizophrenic patients [87], supporting its postulated role in the outbreak of the disease [88]. However, our data were in contrast with these findings, as we found a significant increase in *Psd95* mRNA levels both in animals that experienced isolation only during adolescence or lifelong isolation. Animals isolated only during adulthood did not show any modulation. In contrast, reduced PSD95 expression has been found in the PFC of female rats exposed to social isolation rearing condition [89].

Furthermore, dysfunctions in the GABAergic transmission have been observed following exposure to chronic stress as well as in association with major depression disorder (MDD) and schizophrenia [44, 90]. The synthesis of GABA mainly relies on the enzyme Gad67, whose level is reduced in schizophrenic and MDD patients along with chronically stressed animals [90, 91]. Such a decrease is predominant in neurons expressing the calcium-binding protein parvalbumin (PVB), which is accordingly decreased in such pathological outcomes [45]. However, we did not find any modulation of *Gad67* and *Pvb* expression following our stress procedure, in contrast with a previous study showing a significant down-regulation of *Pvb* after three weeks of social deprivation at adulthood [92]. Although the expression of *Pvb* was not impacted by isolation rearing conditions, we found that hyperactive animals expressed lower *Pvb*, confirming the fact that impairments in the GABAergic system are coupled with a hyperactive schizophrenic-like phenotype.

Considering the role of dopamine in schizophrenia, we found limited changes in D1 and D2 receptors following SI during adolescence, with a trend toward a decreased expression, whereas we observed a consistent elevation of D2 mRNA levels after social isolation at adulthood. These changes are in line with a previous report showing that chronic stress exposure may increase *Drd2* levels [93, 94] and suggest a complex regulation of this system based on the timing of the adverse experience.

Both behavioral and molecular data indicate that AdSI might be a suitable candidate for refining social isolation experiments. AdSI showed comparable hyperactivity, no long-lasting impact on the wellbeing parameter, and most molecular features of SI. However, we would like to emphasize that this observation might be strain- or sex-specific. Since male rats are generally more susceptible to SI outcomes [1] and due to technical limitations in the lab, we focused on male rats in this study. Furthermore, we concentrated the analysis for psychiatry-related parameters to the novelty induced exploration, since this a robust marker for the isolation syndrome. We decided to limit our analysis on locomotor hyperactivity as an indicator to prevent frequent handling, as this could confound the outcome [28]. For other research questions different parameters, e.g. sensory gating might be relevant as well.

In terms of welfare, there is growing evidence that multimodal analysis is a more valuable approach towards evidence-based assessment of severity in laboratory animals [95–97]. In this study, we focused on burrowing and could not find differences between the groups besides the short-term decrease directly after resocialization. More studies on other relevant parameters like nesting, corticosterone release, saccharine preference, grimace scale, or heart rate variability are necessary to fully comprehend the potential benefit of AdSI over SI.

In summary, our study demonstrates that the deprivation of social contacts, at any time of life in laboratory rats, can firmly impair neuroplasticity processes within the prefrontal cortex. These findings have strong translational value, suggesting that an isolation housing condition could be detrimental not only if experienced during the first stages of life but also later on. Moreover, we established that some deficits induced by adolescent stress are long-lasting since they were not attenuated after a period of social housing. Surprisingly, we could not find altered wellbeing due to the isolation housing per se. We observed only a short-term effect after resocialization. Although some evidence suggests that resocialization might normalize isolation-induced parameters, the regrouping acts as a temporary stressor. This should be taken into consideration when changing social groups during experimental procedures. The shift of the isolation period from the more susceptible adolescence phase to the adult phase could be an adequate refinement measure for research questions. It is important to approach the refinement of animal experiments whenever possible and consider potential improvements for the subjects.

## Supporting information

S1 Data Raw data burrowing long format. (XLSX)

S2 Data Raw data burrowing. (XLSX)

S3 Data Raw data mRNA. (XLSX)

S4 Data Raw data open field. (XLSX)

S5 Data Raw Data Z scores. (XLSX)

S1 Table Primers and probes assays. (DOCX)

S1 File The ARRIVE essential 10: Author checklist. (PDF)
