# Correction: Social isolation in rats: Effects on animal welfare and molecular markers for neuroplasticity

**DOI:** 10.1371/journal.pone.0248070

**Published:** 2021-02-26

**Authors:** Veronica Begni, Alice Sanson, Natascha Pfeiffer, Christiane Brandwein, Dragos Inta, Steven R. Talbot, Marco Andrea Riva, Peter Gass, Anne Stephanie Mallien

The eighth author, Peter Gass, is incorrectly noted as the corresponding author. The correct corresponding author is Anne Stephanie Mallien. Anne Stephanie Mallien’s email address is: anne.mallien@zi-mannheim.de
