# Supplementary material for: Social isolation in rats: Effects on animal welfare and molecular markers for neuroplasticity
Source: PLoS One. 2020 Oct 27;15(10):e0240439. doi: 10.1371/journal.pone.0240439 (PMC7591026; doi:10.1371/journal.pone.0240439)
Supplement: S1 Table — (DOCX) [file pone.0240439.s006.docx]

| **Gene** | **Forward Primer** | | **Reverse Primer** | **Probe** |
| --- | --- | --- | --- | --- |
| *Bdnf tot* | 5’-AAGTCTGCATTACATTCCTCGA-3’ | | 5’GTTTTCTGAAAGAGGGACAGTTTAT-3’ | 5’-TGTGGTTTGTTGCCGTTGCCAAG-3’ |
| *Arc* | 5’-GGTGGGTGGCTCTGAAGAAT-3’ | | 5-ACTCCACCCAGTTCTTCACC-3’ | 5’-GATCCAGAACCACATGAATGGG-3’ |
| *Gad67* | 5’-ATACTTGGTGTGGCGTAGC-3’ | | 5’-AGGAAAGCAGGTTCTTGGAG-3’ | 5’-AAAACTGGGCCTGAAGATCTGTGGT-3’ |
| *Psd95* | 5’-CAAGAAATACCGCTACCAAGATG-3’ | | 5’-CCCTCTGTTCCATTCACCTG-3’ | 5’-TCAACACGGACACCCTAGAAGCC-3’ |
| *Pvb* | 5’-CTGGACAAAGACAAAAGTGGC-3’ | | 5’-GACAAGTCTCTGGCATCTGAG-3’ | 5’-CCTTCAGAATGGACCCCAGCTCA-3’ |
| **Gene** | **Assay ID** | |  |  |
| *Bdnf long 3’-UTR* | | Rn02531967_s1 |  |  |
| *Bdnf transcript IV* | | Rn01484927_m1 |  |  |
| *Bdnf transcript VI* | | Rn01484928_m1 |  |  |

**S1 Table:** Primers and probes assays
